# Surgical Outcomes of Hugo™ RAS Robot-Assisted Partial Nephrectomy for Cystic Renal Masses: Technique and Initial Experience

**DOI:** 10.3390/jcm13123595

**Published:** 2024-06-19

**Authors:** Francesco Prata, Andrea Iannuzzi, Francesco Tedesco, Alberto Ragusa, Angelo Civitella, Matteo Pira, Marco Fantozzi, Leonilde Sica, Roberto Mario Scarpa, Rocco Papalia

**Affiliations:** Department of Urology, Fondazione Policlinico Universitario Campus Bio-Medico, 00128 Rome, Italy; f.prata@policlinicocampus.it (F.P.); francesco.tedesco@unicampus.it (F.T.); alberto.ragusa@unicampus.it (A.R.); a.civitella@policlinicocampus.it (A.C.); matteo.pira@unicampus.it (M.P.); marco.fantozzi@unicampus.it (M.F.); l.sica@policlinicocampus.it (L.S.); r.scarpa@policlinicocampus.it (R.M.S.); rocco.papalia@policlinicocampus.it (R.P.)

**Keywords:** Hugo RAS system, off clamp, partial nephrectomy, cystic renal masses, minimally invasive

## Abstract

**Background**: The Hugo™ Robot-Assisted Surgery (RAS) system is a new cutting-edge robotic platform designed for clinical applications. Nevertheless, its application for cystic renal tumors has not yet been thoroughly investigated. In this context, we present an initial series of Robot-Assisted Partial Nephrectomy (RAPN) procedures carried out using the Hugo™ RAS system for cystic renal masses. **Methods**: Between October 2022 and January 2024, twenty-seven RAPN procedures for renal tumors were performed at Fondazione Policlinico Universitario Campus Bio-Medico. Our prospective board-approved dataset was queried for “cystic features” (n = 12). Perioperative data were collected. The eGFR was calculated according to the CKD-EPI formula. Post-operative complications were reported according to the Clavien–Dindo classification. Computed tomography (CT) scans for follow-up were performed according to the EAU guidelines. Trifecta was defined as the coexistence of negative surgical margin status, no Clavien–Dindo grade ≥ 3 complications, and eGFR decline ≤ 30%. **Results**: All the patients successfully underwent RAPN without the need for conversion or additional port placement. The median docking and console time were 5.5 (IQR, 4–6) and 79.5 min (IQR, 58–91 min), respectively. No intraoperative complications occurred, as well as clashes between instruments or with the bedside assistant. Two minor postoperative complications were recorded (Clavien–Dindo II). At discharge, serum creatinine and eGFR were comparable to preoperative values. Only one patient (8.4%) displayed positive surgical margins. The rate of trifecta achievement was 91.7%. **Conclusions**: RAPN for cystic renal masses using the novel Hugo™ RAS system can be safely and effectively performed. This robotic system provided satisfactory peri-operative outcomes, preserving renal function and displaying low postoperative complications and a high trifecta rate achievement.

## 1. Introduction

The prevalence of renal cell carcinoma (RCC) is relatively limited, comprising just 3% of all malignant tumors. Interestingly, most of the RCC cases are incidentally identified during imaging studies conducted for unrelated medical conditions. Furthermore, cystic RCCs represent a relatively uncommon subtype, constituting 2.5–12% of all RCCs [1].

Nowadays, distinguishing between benign and malignant cystic masses has become significantly more straightforward, attributed to the introduction of the Bosniak renal cyst classification system and advancements in diagnostic imaging technology. Bosniak I and II cysts are considered benign and typically do not necessitate follow-up. Bosniak IV cysts, predominantly (83%) malignant tumors, exclusively exhibit pseudo-cystic changes. Managing Bosniak IIF and III cysts poses challenges for clinicians [2,3,4].

The preferred approach for treating renal masses is partial nephrectomy (PN) whenever technically feasible [2,5]. Cystic renal tumors pose surgical challenges due to the risk of rupturing the cyst wall during tumor removal, which could lead to local tumor spillage and a theoretical risk of tumor recurrence. This concern may cause many surgeons to prefer traditional open surgery over a minimally invasive approach. However, there is limited evidence regarding the actual risk of tumor recurrence following cyst wall rupture [6]. Although laparoscopic PN (LPN) has been demonstrated as a feasible option for cystic renal tumors, it requires advanced surgical expertise to reduce the risk of cyst rupture and localized exudation. In recent years, numerous studies indicate that robot-assisted partial nephrectomy (RAPN) has a lower learning curve, even for surgeons without prior laparoscopic experience. The advantages of the robotic approach include shorter operation times, less blood loss, reduced warm ischemia time (WIT), and the improved preservation of the remaining kidney function [7,8]. Furthermore, RAPN has proven successful in handling complex renal tumors [9].

Following the introduction of the initial Da Vinci platform, the subsequent generations of robotic systems with advanced technical enhancements have emerged as alternative solutions. These innovations aim to overcome the inherent platform limitations, expand the surgical indications, and result in comprehensive cost reductions, potentially increasing the accessibility to robotic surgery. One such system is the Hugo™ RAS (Medtronic, Minneapolis, MN, USA©), designed as a multiport robotic system featuring four independent arm-carts, an “open” console, three-dimensional (3D) high-definition glasses with a head-tracking safety system, and gun-like ergonomic controllers. The key technical advantages include a more extensive working space for the bedside assistant, improved trocar positioning for better ergonomics, and overall cost-effectiveness [10,11].

However, its application for cystic renal tumors has not been explored yet. In this context, we present an initial series of off-clamp RAPN procedures conducted using the Hugo™ RAS system for cystic renal masses.

## 2. Materials and Methods

### 2.1. Patient Population

From October 2022 to January 2024, Fondazione Policlinico Universitario Campus Bio-Medico, a high-volume center for off-clamp PN, conducted twenty-seven RAPN procedures for renal tumors. A search in our prospectively approved dataset was conducted specifically for cases with “cystic features”, identifying twelve such instances (*n* = 12). All the patients participating in the study provided written informed consent. Baseline and perioperative data were systematically collected for analysis. Additionally, all the subjects underwent pre-operative urine culture and imaging via computed tomography (CT) scans, with renal masses being classified based on the R.E.N.A.L. score [12].

### 2.2. Endpoints and Data and Statistical Analysis

The primary endpoint was to evaluate the feasibility and safety of this new platform in the off-clamp RAPN for cystic renal masses. Post-operative complications were reported according to the Clavien–Dindo classification [13]. The body mass index (BMI) was calculated as the weight in kilograms (kg) divided by the height in meters (m), squared (kg/m^2^), and the estimated glomerular filtration rate (eGFR) was calculated according to the Chronic Kidney Disease Epidemiology Collaboration (CKD-EPI) formula. Trifecta was defined as the coexistence of negative surgical margin status, no Clavien–Dindo grade ≥ 3 complications, and an eGFR decline ≤ 30%. Continuous variables are presented as median and interquartile ranges (IQRs) while frequencies are used to report the categorical variables. STATA (StataCorp. 2021. Stata Statistical Software: Release 17. College Station, TX, USA: StataCorp LLC) was used for the statistical analyses.

### 2.3. Trocar Placement, Docking System, and Surgical Procedure

Trocar placement and docking setting were performed using our described technique [14].

We utilized a modified extended flank position by placing the patient at the edge of a surgical bed and using a moderate 45° flexion to increase the space between the homolateral iliac spine and the rib margin. The initial robotic trocar (11 mm, endoscope port) was inserted transperitoneally along the pararectal line, about 14 cm below the xiphopubic line. We then placed up to three additional 8 mm robotic ports at least 8 cm laterally from the camera port while maintaining a 2 cm safety margin from any bone prominences. Two further laparoscopic ports (12 mm) for the bedside assistant were positioned medially, approximately 8 cm from the robotic ports, to prevent interference with the robotic instruments. The bedside assistant’s position, whether standing or seated, varied based on the patient’s anatomical characteristics, the height of the surgical bed, and the angles of the robotic arm docking and tilt. The primary surgeon was seated during the operation to optimally manage the Hugo™ RAS controllers. The AirSeal™ system (SurgiQuest, Milford, CT, USA©) was used to induce pneumoperitoneum, maintaining a standard intra-abdominal pressure of 12 mmHg. The recommended trocar configuration for the Hugo™ RAS system typically includes four robotic arms, consisting of an 11 mm optic port and three 8 mm robotic instrument ports. This standard setup allows room for just one laparoscopic trocar for the bedside assistant. However, in our surgical arrangement, we employed three to four robotic arms with a three-instrument configuration and allocated two 12 mm laparoscopic trocars for the bedside assistant. The choice between the three- or four-robotic arm configuration was based on the lead surgeon’s preference, leading to a different degree of involvement of the bedside assistant in the surgical procedure, reflecting the modularity of the Hugo™ RAS platform. Before docking, arm carts were positioned 45 to 60 cm away from the patient, with three arm carts behind the patient’s back, the fourth arm in front, and the energy tower at the bottom of the bed. The docking and tilt angles varied according to the side of the lesion. This new setup was used to perform the standard RAPN for solid lesions, and the same setup was adopted for tumors with cystic features, with no technical differences in trocar placement and docking settings.

The first surgeon, bedside assistants, and scrub nurses participating in the operations had all undergone technical training on the Hugo™ RAS system provided by Medtronic at the ORSI Academy in Aalst, Belgium. A single surgeon (R.P.), with extensive experience in minimally invasive PN and off-clamp techniques through a conventional trans-peritoneal route, performed all the procedures. The surgical setup involved the use of monopolar curved shears, a fenestrated grasper, and a large needle driver in a three-instrument configuration.

The key steps of the clampless procedures included making an incision along the Toldt line, mobilizing the kidney, isolating it from the pre-renal fat tissue, and adopting a direct approach to the renal mass. Bleeding from the resection bed was managed using monopolar energy, and renorrhaphy was carried out using a 2/0 Monocryl single-running suture employing a sliding-clips technique. Hemostasis was further enhanced by applying hemostatic agents on the renal rim (TABOTAMP fibrillar™ and TachoSil^®^). After ensuring the normotensive control of hemostasis, the closure of Gerota’s fascia and placement of a drain concluded the procedure.

### 2.4. Follow-up Schedule

After the surgery, a follow-up schedule was implemented, consisting of the following assessments: laboratory examinations, encompassing blood panel, electrolytes, and renal function at 1 month post-surgery and then every three months, while an abdominal CT scan was performed after 3 months from the intervention and then every six months.

## 3. Results

All the patients successfully underwent RAPN without the need for conversion or additional port placement. Baseline and demographic data are presented in Table 1. The median age and BMI were 68.5 years (IQR, 62–72) and 27.3 kg/m^2^ (IQR, 26.4–28.2), respectively. The median tumor size and R.E.N.A.L. score were 47.5 mm (IQR, 34–55) and 7 (IQR, 5–9), respectively. The pre-operative serum creatinine and eGFR were 0.98 mg/dL (IQR, 0.81–1.17) and 69.5 mL/min/1.73 m^2^ (IQR, 47.5–74.3), respectively. The perioperative data are reported in Table 2. The median docking and console time were 5.5 (IQR, 4–6) and 79.5 min (IQR, 58–91 min), respectively. The Median Estimated Blood Loss (EBL) was 200 mL (IQR, 100–500 mL). No intraoperative complications occurred, nor clashes between instruments or with the bedside assistant. None of the cystic masses experienced any rupture during the procedures. Two minor post-operative complications were recorded (Clavien–Dindo II). The median length of stay (LOS) was 4 days (IQR, 3–5 days). At discharge, the serum creatinine level and eGFR were comparable to the preoperative values. The primary histology was cystic clear cell renal cell carcinoma (ccRCC) in six cases (50%) and cystic papillary RCC in the remaining six patients (50%). Only one patient (8.4%) displayed positive surgical margins. The rate of trifecta achievement was 91.7%. The median follow-up was 10 months. The median hemoglobin, creatinine, and eGFR at 1 month follow-up were 12 (10–14), 0.93 (0.86–0.97), and 79.9 (66.2–86.9), respectively. The median hemoglobin, creatinine, and eGFR at the last follow-up were 13 (11–14), 0.86 (0.83–1.03), and 80.1 (70.4–86.9), respectively. The CT scans at 3 months showed no signs of persistence or recurrence of disease.

## 4. Discussion

PN is considered the gold standard treatment for organ-confined RCC. It should be contemplated whenever technically feasible, aiming to preserve as much healthy renal parenchyma as possible to minimize the impact on renal function [2].

The role of RAPN in the management of renal masses has experienced exponential growth over the past decade. Several studies have consistently highlighted the technical advantages of robot-assisted vs. pure laparoscopic or open techniques, emphasizing its proficiency in tumor excision and suturing. Indeed, robotic surgery has proven to offer enhanced operative stability and clearer views, thereby facilitating the dissection and intraoperative hemostasis. Therefore, its capacity to yield improved renal functional outcomes has solidified its reputation as an excellent tool for urologists [15,16,17,18].

Moreover, with the maturation of surgical expertise, there has been a notable expansion in the indications for RAPN, now encompassing more challenging and larger masses [19,20,21].

Compared with solid tumors, performing minimally invasive PN for cystic renal masses presents a significant challenge due to the necessity for advanced surgical proficiency, as there is an elevated risk of cyst wall rupture and subsequent tumor spillage.

In a recent report, Novara et al. demonstrated that RAPN for cystic renal tumors was associated with a lower operative time compared to solid tumors, but stratifying via the PADUA score, such an association was evident in the intermediate and high-risk category, not in the low-risk one. No statistically significant difference was shown in regard to the EBL, postoperative complications, and eGFR decline. Yagisawa et al. showed a longer operative time in RAPN for cystic renal tumors compared to solid ones, but after a propensity 1:1 score matching, this difference was not statistically significant anymore [22]. Differently, Abdel Raheem et al. showed a longer operative time in solid tumors compared to cystic ones with no statistically significant difference in the peri- and postoperative complications, especially in the eGFR decline at 1, 3, and 6 months [1]. 

However, it is noteworthy that the existing data in the literature are conflicting, highlighting the necessity for additional studies to provide clarity on this matter. In a recent series, Xu et al. demonstrated that the intraoperative rupture of malignant cystic renal masses negatively impacted the oncological outcomes. The estimated recurrence-free survival (RFS), metastasis-free survival (MFS), and cancer-specific survival (CSS) were found to be shorter in the cyst ruptured group compared to non-ruptured cases (*p* < 0.001; *p* = 0.001; *p* < 0.001). Cox regression analysis revealed that cyst rupture (CR) was an independent prognostic factor for RFS (HR = 7.354; 95% CI = 1.839–29.413; *p* = 0.005), MFS (HR = 8.069; 95% CI = 1.804–36.095; *p* = 0.006), and CSS (HR = 9.643; 95% CI = 2.183–42.599; *p* = 0.003) [23]. Moreover, Chen et al. demonstrated that patients with cyst rupture experienced worse 5-year RFS and 5-year CFS compared to those without cyst rupture (*p* = 0.006 and 0.003, respectively). In addition, multivariate Cox analysis revealed that intraoperative cyst rupture was an independent risk factor for both 5-year RFS (*p* = 0.039) and 5-year CFS (*p* = 0.013) [24]. On the contrary, Pradere et al. argued that cyst rupture during partial PN has minimal oncological impact. Their findings showed that the estimated RFS did not exhibit significant differences between patients with and without intraoperative cyst rupture, measuring 100% versus 92.7% at 5 years (*p* = 0.200) [25]. In our series, there were no instances of cystic mass rupture, and upon the initial follow-up CT scan, no patient exhibited disease recurrence. It is important to note that this finding is constrained by the relatively short follow-up period.

There is a scarcity of studies in the existing literature that have conducted comparisons of the outcomes in PN for cystic tumors between robotic and laparoscopic approaches. Recently, Tang et al. reported that RAPN exhibited lower EBL compared to both open and laparoscopic approaches. However, data on LOS, complications, outcomes, recurrence rate, and cancer-specific survival remain inconclusive [6]. Conversely, Calpin et al. found no statistically significant differences in ischemia time, intraoperative complications, positive surgical margins, and trifecta rate between open, laparoscopic, and robotic PN. However, RAPN and laparoscopic partial nephrectomy (LPN) demonstrated lower postoperative complications and shorter LOS compared to open partial nephrectomy (OPN). Additionally, RAPN and LPN were associated with reduced EBL compared to OPN, with RAPN showing superior overall outcomes [26]. In contrast, a recent series by Wurnschimmel et al. reported longer operative times in RAPN compared to LPN, primarily due to the surgical technique applied, which involved inducing selective ischemia in RAPN procedures and total ischemia isolating the renal hilum in LPN procedures [27]. In a retrospective propensity-score-matched study by Chang K.D. et al., open, laparoscopic, and robotic PN were compared among 1308 patients. Over a median 5-year follow-up, similar oncological outcomes were found across the approaches, with comparable rates of local recurrence (*p* = 0.882), distant metastasis (*p* = 0.816), and cancer-related deaths (*p* = 0.779). In terms of perioperative outcomes, RAPN exhibited superiority over open partial nephrectomy and LPN, demonstrating lower EBL (*p* = 0.040 vs. 0.025, respectively) and a shorter hospital stay (*p* = 0.008). Moreover, a significantly lower incidence of chronic kidney disease (CKD) upstaging was observed in the RAPN group compared with LPN (20.55% vs. 32%; *p* = 0.035) and the open approach (20.5% vs. 33.6%; *p* = 0.038). Additionally, the 5-year CKD-free survival rate was significantly higher in the RAPN group (78.4%) compared with the LPN (58.8%) and open partial nephrectomy (65.8%) groups (log-rank *p* = 0.030) [28].

The findings in our series also underscore the safety of this procedure, along with the achievement of excellent functional results. The median estimated blood loss was 200 mL (IQR, 100–500 mL), with no intraoperative complications reported. Only two minor postoperative complications were recorded; indeed, two patients experienced postoperative fever, necessitating the initiation of antibiotic therapy. The median length of stay was 4 days (IQR, 3–5 days). Upon discharge, the serum creatinine level and estimated glomerular filtration rate were comparable to the preoperative values. The median hemoglobin, creatinine, and eGFR at 1 month follow-up were 12 (10–14), 0.93 (0.86–0.97), and 79.9 (66.2–86.9), respectively. The median hemoglobin, creatinine, and eGFR at the last follow-up were 13 (11–14), 0.86 (0.83–1.03), and 80.1 (70.4–86.9), respectively.

To the best of our knowledge, this represents the first series of off-clamp RAPN procedures for cystic renal masses performed using the Hugo™ RAS system. The median docking time was limited to 5.5 min (IQR, 4–6 min), and the median console time was 79.5 min (IQR, 58–91 min). There were no instances of instrument clashing, and therefore, no revision of the surgical configuration was required. No complications occurred during the surgery, affirming the feasibility and safety of the novel Hugo RAS system. Several assumptions can be made regarding the satisfactory outcomes of our RAPN series and the absence of cystic rupture. Firstly, the unique setup that we adopted, facilitated via the adaptability of the platform, meets various procedural needs and provides greater comfort during high-precision surgical procedures. Indeed, the Hugo™ RAS system, developed by Medtronic, has emerged as a key alternative to the standard Da Vinci system. This new RAS platform is notable for its improved modularity, thanks to its design of separate arm carts, which can potentially reduce the docking time and minimize the likelihood of accidental clashes between robotic and laparoscopic instruments during surgery. These enhancements are particularly important in RAPN surgeries, where seamless collaboration between the lead surgeon and the bedside assistant is crucial to minimizing the risk of significant intraoperative bleeding or tumor rupture.

To our knowledge, this study represents the first series of off-clamp RAPN procedures for cystic renal masses performed using the Hugo™ RAS system. The median docking time was restricted to 5.5 min (IQR, 4–6 min), and the median console time was 79.5 min (IQR, 58–91 min). No instances of instrument clashing occurred, thus avoiding the need for surgical configuration revisions. Importantly, no complications were encountered during surgery, confirming the feasibility and safety of the innovative Hugo RAS system.

Several conclusions can be drawn from the successful outcomes of our RAPN series and the absence of cystic rupture. Firstly, the unique setup that we employed, facilitated by the platform’s adaptability, accommodates diverse procedural requirements and enhances comfort during precise surgical procedures. Indeed, the Hugo™ RAS system, developed by Medtronic, presents a significant alternative to the standard Da Vinci system. Notably, this new RAS platform offers enhanced modularity with separate arm carts, potentially reducing the docking time and minimizing the risk of inadvertent clashes between robotic and laparoscopic instruments during surgery. These improvements are particularly crucial in RAPN surgeries, where seamless collaboration between the lead surgeon and the bedside assistant is paramount in minimizing intraoperative bleeding or tumor rupture. The controllers feature ergonomic “pistol-like” grips that provide superior control, and which are particularly beneficial for delicate tasks. Additionally, the system’s “trigger” mechanism enhances the stability and reduces the strain on the surgeon’s hand and wrist. Effective communication between the lead surgeon and the surgical team is essential for the success of RAPN, and the Hugo™ RAS system facilitates this through its non-immersive console, enabling clear instructions and coordination. Combined with the ergonomic instrument design and advanced console functionality, these features aim to enhance the intraoperative efficiency, manage unexpected bleeding more effectively, and reduce the risk of adverse incidents that could harm patients. Another significant aspect of our approach is the enhanced role of the bedside assistant, enabled by the use of two laparoscopic instruments. This setup allows for the simultaneous use of two surgical suctions with irrigation, improving the visualization of tumor borders during enucleation and precise differentiation between healthy renal parenchyma and the renal mass [29]. 

Despite these advancements, we acknowledge several limitations in this study. Foremost among these is its single-center design, which limits the diversity of the patient demographics and surgical practices that could influence outcomes. Additionally, the relatively small sample size and short follow-up duration are significant constraints, potentially compromising the robustness and generalizability of our findings. Moreover, while the Hugo™ RAS system has shown promising outcomes and features an efficient docking system, it is critical to note that all the procedures were performed exclusively by a highly specialized team experienced in off-clamp laparoscopic PN. This expertise may not be representative of broader surgical settings, raising questions about the applicability of our results to other centers with varying levels of surgical proficiency. Therefore, our results should be interpreted cautiously and validated through larger-scale studies involving diverse patient populations and longer-term follow-up. Further research is urgently needed to establish standardized surgical protocols for off-clamp RAPN using the Hugo™ RAS system and to define its specific role and effectiveness in advancing the field of robotic kidney surgery.

## 5. Conclusions

RAPN for cystic renal masses utilizing the innovative Hugo™ RAS system has been demonstrated to be both safe and effective. This advanced robotic system exhibited satisfactory peri-operative outcomes, significantly contributing to the preservation of renal function. The surgical procedures not only resulted in low incidences of postoperative complications but also achieved a high rate of the trifecta outcome, which includes negative surgical margins, minimal renal functional decline, and no perioperative complications. This impressive performance underscores the overall success and reliability of this surgical approach.

## Figures and Tables

**Table 1:** Baseline and demographic data of the patient cohort.

Variable	Cohort (*n* = 12)
**Age (*n*, median, IQR)** **Gender (*n*, %)** **Male** **Female**	68.5 (62–72) 6 (50%) 6 (50%)
**BMI (kg/m^2^, median, IQR)**	27.3 (26.40–28.20)
**ASA score (*n*, %)** **I** **II** **III** **IV**	2 (16.7%) 10 (83.30%) 0 (0%) 0 (0%)
**Charlson Comorbidity Index (median, IQR)**	4 (4–5)
**Diabetes (*n*, %)**	2 (16.70%)
**Hypertension (*n*, %)**	6 (50%)
**Preoperative Hemoglobin (g/dL, median, IQR)**	13.6 (13–14.70)
**Preoperative Creatinine (mg/dL, median, IQR)**	0.98 (0.81–1.17)
**Preoperative eGFR (mL/min/1.73 m^2^, median, IQR)**	69.5 (47.50–74.30)
**Clinical Tumor Size (mm, median, IQR)**	47.5 (34–55)
**cT (*n*, %)** **T1a** **T1b** **T2a**	4 (33.30%) 6 (50%) 2 (16.70%)
**Side (*n*, %)** ** Right** **Left **	6 (50%) 6 (50%)
**R.E.N.A.L. score (median, IQR)**	7 (5–9)

BMI: body mass index; ASA: American Society of Anesthesiologists; eGFR: estimated glomerular filtration rate.

**Table 2:** Perioperative and postoperative data of the patient cohort.

Variable	Cohort (*n* = 12)
**Docking Time (min, median, IQR)**	5.5 (4–6)
**Console Time (min, median, IQR)**	79.5 (58–91)
**Estimated blood loss (mL, median, IQR)**	200 (100–500)
**Perioperative complications (*n*, %)**	2 (16.70%)
**Length of stay (days, median, IQR)**	4 (3–5)
**Hemoglobin at discharge (g/dL, median, IQR)**	11 (8.90–11.90)
**Creatinine at discharge (mg/dL, median, IQR)**	0.91 (0.82–1.12)
**eGFR at discharge (mL/min/1.73 m^2^, median, IQR)**	67.9 (63.50–69.60)
**Clavien Dindo Complications (*n*, %)** - **I** - **II** - **III** - **IV**	0 (0%) 2 (16.70%), fever 0 (0%) 0 (0%)
**Pathological Size (mm, median, IQR)**	41.5 (25–55)
**Histology_subtype (*n*, %)** - **Cystic Clear Cell RCC** - **Cystic Papillary RCC**	6 (50%) 6 (50%)
**Positive Margins (*n*, %)**	1 (8.40%)
**pT Stage (*n*, %)** - **1a** - **1b** - **2a** - **2b** - **3a**	6 (50%) 2 (16.70%) 2 (16.70%) 1 (8.40%) 1 (8.40%)
**Last follow-up (months, median, IQR)**	10 (3–12)
**Hemoglobin at 1 month (g/dL, median, IQR)**	12 (10–14)
**Creatinine at 1 month (mg/dL, median, IQR)**	0.93 (0.86–0.97)
**eGFR at 1 month (mL/min/1.73 m^2^, median, IQR)**	79.9 (66.20–86.90)
**Hemoglobin at the last follow-up (g/dL, median, IQR)**	13 (11–14)
**Creatinine at the last follow-up (mg/dL, median, IQR)**	0.86 (0.83–1.03)
**eGFR at the last follow-up (mL/min/1.73 m^2^, median, IQR)**	80.1 (70.40–86.90)
**Trifecta achievement rate (*n*, %)**	11 (91.70%)

eGFR: estimated glomerular filtration rate.

## Data Availability

The original contributions presented in the study are included in the article, further inquiries can be directed to the corresponding author.
